# SCIT with a high-dose house dust mite allergoid is well tolerated: safety data from pooled clinical trials and more than 10 years of daily practice analyzed in different subgroups

**DOI:** 10.1007/s40629-018-0059-x

**Published:** 2018-07-25

**Authors:** Ludger Klimek, Gabriele-Cornelia Fox, Susanne Thum-Oltmer

**Affiliations:** 1 Center for Rhinology and Allergology, An den Quellen 10, 65183 Wiesbaden, Germany; 2 Allergopharma GmbH & Co. KG, Reinbek bei Hamburg, Germany

**Keywords:** Tolerability, Subcutaneous allergen immunotherapy, Allergic rhinitis, Asthma, Children

## Abstract

**Background:**

Efficacy of house dust mite (HDM) allergen immunotherapy (AIT) in allergic rhinitis and controlled allergic asthma has been documented in controlled trials with adults and children. However, tolerability comparing clinical development and post marketing data, particularly in different subgroups, is missing.

**Methods:**

We performed an analysis of pooled safety data for subcutaneous AIT (SCIT) with a high-dose house dust mite allergoid from 6 randomized, controlled trials (RCT) in HDM allergic respiratory disease (ARD) and of post marketing safety data from more than 10 years including different subgroups (age, gender, asthma status).

**Results:**

In all, 500 patients with ARD were treated in RCTs: 279 received the marketed dose of 1800 protein nitrogen units (PNU) high-dose HDM allergoid AIT (214 double-blind placebo controlled [HDM-DBPC], 65 children/adolescents usual care controlled [HDM-RCT(UC)]), and 221 placebo (PL). 38.8% adverse events (AEs) were observed with 1800 PNU in HDM-DBPC (31.2% PL, 35.5% HDM-ALL [1800 PNU]); the difference was primarily because of local reactions; there was no difference in systemic reactions (10.9% PL, 11.2% HDM-DBPC, 11.2% HDM-ALL); one out of 279 high-dose HDM allergoid-treated patients had a serious adverse event (SAE).

Children (*n* = 39)/adolescents (*n* = 26) had fewer related AEs and local reactions compared to adults; systemic reactions: children 12.8%, adults 11.2% adolescents 7.7%. Females had slightly more AEs. Treatment was well tolerated in asthmatic patients (*n* = 267; GINA I *n* = 32, II *n* = 104, III *n* = 17, 114 no classification).

In more than 10 years more than 100,000 patients were treated with high-dose HDM allergoid (1800 PNU) under daily practice conditions. Adverse drug reactions (ADRs) were reported in 0.5% of patients. 94.6% of these ADRs were expected.

**Conclusion:**

SCIT with the marketed dose of high-dose HDM allergoid was well tolerated in clinical development and in daily practice. There was no increased risk for the investigated patient subgroups. Tolerability was comparable to HDM sublingual immunotherapy (SLIT) tablets.

## Introduction

Allergen immunotherapy (AIT) is a therapy with disease-modifying effects and the only available treatment to target the disease instead of the symptoms. By administering allergen extracts, specific blocking antibodies, tolerance-inducing cells, and mediators are activated. These prevent further exacerbation of the allergen-triggered immune response, block the specific immune response, and attenuate the inflammatory response in tissue [1, 2].

House dust mite (HDM) allergy is strongly implicated in the pathogenesis of respiratory allergic disease and a large proportion of patients with allergic rhinitis (AR), allergic asthma (AA), or both, are sensitized to HDM, predominantly *Dermatophagoides pteronyssinus* and *Dermatophagoides farinae* [3].

Efficacy of AIT in HDM allergy is documented by a number of controlled trials in adults and few controlled trials in children [1, 4]. Subcutaneous immunotherapy (SCIT) has been well investigated for individual preparations in controlled bronchial asthma as defined by the Global Initiative for Asthma (GINA) 2007 as well as in intermittent and mild persistent asthma (GINA 2005). AIT is recommended as a treatment option, in addition to allergen avoidance and pharmacotherapy in GINA 2017, provided there is a clear causal link between respiratory symptoms and the relevant allergen [1, 4, 5].

The high-dose HDM allergoid is a depot formulation with aluminum hydroxide for subcutaneous AIT. Formaldehyde and glutaraldehyde have been used in combination to chemically modify an aqueous extract of purified HDM bodies and to produce a stable allergoid. The allergen extract consists of the HDM *Dermatophagoides pteronyssinus *or *Dermatophagoides farinae* alone or in combination. The product is manufactured at two different strengths, which enables a low initial dose and increasing concentration of allergens in the further course of treatment. In maintenance therapy, intervals of up to 8 weeks are very convenient and allow for flexibility. The marketed dose is 3000 protein nitrogen units (PNU)/mL in strength B (strength A: 300 PNU/mL obtained by a 1:10 dilution of strength B) with a maximum injection volume of 0.6 ml (1800 PNU; [8]). The major allergen content of the high-dose HDM allergoid *D. pteronyssinus is* 12 μq/ml Der p 1 and 10 μg/ml Der p 2; for the high-dose HDM allergoid *D. farinae it is *20 μg/ml Der f 1 and 15 μg/ml Der f 2 [9].

This HDM SCIT product has been shown to be effective in patients with mite-induced allergic asthma. Adding the mite allergoid SCIT to pharmacologic treatment was an effective and safe strategy to reduce corticosteroid doses while maintaining disease control and significantly improved bronchial allergen provocation (BAP; [6, 7]). There is however no data available comparing safety information for AIT products from clinical development and spontaneous safety reports from daily practice since launch, particularly not in different patient subgroups [10].

The publication presents safety data of this high-dose HDM allergoid in clinical development and from adverse drug reactions (ADRs) spontaneously reported after launch, including subgroup analysis. SLIT is reported as having ADRs in 40–75% of the cases as temporary local mucosal reactions (pruritus or dysesthesia in the oral cavity, swelling of the oral mucosa, throat irritation) and considered as having a better safety profile than SCIT, especially with respect to systemic reactions, anaphylaxis, and other severe systemic reactions [1]. Therefore, the safety data of this product were compared to the safety of two different SLIT mite tablets as benchmark.

## Material and methods

### Product

The high-dose HDM allergoid (Acaroid®) consists of the HDM *Dermatophagoides pteronyssinus *alone (used in Trials AL0104av (EudraCT: 2004-003892-35), 97-09 M, 97-09 UK, AL0106ac (EudraCT: 2006-000934-11), and AL1009ac (EurdraCT: 2011-002248-29)) or in 1:1 combination with *Dermatophagoides farinae* (used in Trial AL0400av; Table 1). 97-09 M, 97-09 UK, and AL0400av were performed before introduction of the EudraCT database. The drug product is a suspension administered by subcutaneous injection.

**Table 1:** Summary of the high-dose HDM allergoid clinical trials included in the pooled safety analysis

Study	Population		Treatment	*n*	Included in safety set of			
	Indication	Age (years)			HDM-ALL	HDM-DBPC	HDM-RCT(UC)	Placebo
					(*n* = 279)	(*n* = 214)	(*n* = 65)	(*n* = 221)
97-09 M^a^	Allergic rhinitis/rhinoconjunctivitis	18–58	1800 PNU	20	Yes	Yes	–	–
			Placebo	20	–	–	–	Yes
97-09 UK^a^	Allergic rhinitis/rhinoconjunctivitis	22–54	1800 PNU	15	Yes	Yes	–	–
			Placebo	15	–	–	–	Yes
AL0106ac EudraCT: 2006-000934-11	Allergic rhinitis/rhinoconjunctivitis	19–46	1800 PNU	51	Yes	Yes	–	–
			Placebo	57	–	–	–	Yes
Al0400av^a^	Allergic rhinitis/rhinoconjunctivitis	18–54	1800 PNU	69	Yes	Yes	–	–
			Placebo	66	–	–	–	Yes
Al1009ac EurdraCT: 2011-002248-29	Controlled allergic bronchial asthma and rhinitis/rhinoconjunctivitis	18–40	600 PNU	24	–	–	–	–
			1800 PNU	31	Yes	Yes	–	–
			3000 PNU	28	–	–	–	–
			5400 PNU	31	–	–	–	–
			Placebo	32	–	–	–	Yes
AL0104av EudraCT: 2004-003892-35	Allergic asthma with or without allergic rhinitis/rhinoconjunctivitis	6–406–40	–	–	–	–	–	–
		≥18	1800 PNU	28	Yes	Yes	–	–
		≥18	Placebo	31	–	–	–	Yes
		<12	1800 PNU	39	Yes	–	Yes	–
		12–17	1800 PNU	26	Yes	–	Yes	–
		6–17	Usual care	32	–	–	–	–

^a^Performed before introduction of the EudraCT database

*HDM* house dust mites, *HDM-DBPC* high-dose HDM allergoid AIT double-blind placebo controlled, *HDM-RCT(UC)* high-dose HDM allergoid AIT usual care controlled, *HDM-ALL* HDM-DBPC plus HDM-RCT(UC), *PNU* protein nitrogen units

The following strengths were used in Trials AL0104av, 97-09 M, 97-09 UK, AL0400av, and AL0106ac: strength B: 3000 PNU/mL; strength A: 300 PNU/mL (obtained by a 1:10 dilution of strength B). In the dose range finding trial AL1009ac 4 different doses were tested: 600 PNU/mL, 1800 PNU/mL, 3000 PNU/mL and 5400 PNU/mL.

### Study population

This publication presents safety information from 6 completed, randomized, controlled clinical trials conducted between 2000 and 2015 in patients with allergic rhinitis/rhinoconjunctivitis (97-09 M, 97-09 UK, AL0400av, and AL0106ac), patients with allergic asthma and allergic rhinitis/rhinoconjunctivitis (Trial AL1009ac), or subjects with allergic asthma with or without allergic rhinitis/rhinoconjunctivitis (Trial AL0104av). Demographic details are shown in Table 2. We present safety information from patients treated with the marketed dose of 1800 PNU (*n* = 279) as maintenance dose (100% *D. pteronyssinus* or 50%/50% *D. pteronyssinus*/*D. farinae* allergens/mixtures). The maximum treatment duration was 3 years and 2 years with placebo, respectively; the maximum double-blind, placebo-controlled treatment phase was 2 years (97-09 M, 97-09 UK, AL0400av, and AL0106ac, AL0104av adults). In study AL0104av, children and adolescents (*n* = 65; 6–17 years) with asthma were treated for up to 3 years with this HDM AIT (Group: HDM-RCT(UC)).

**Table 2:** Demographics and baseline characteristics

	Statistics	97-09 M	97-09 UK	AL0104av	AL0106ac	AL0400av	AL1009ac	Total
		(*n* = 20)	(*n* = 15)	(*n* = 93)	(*n* = 51)	(*n* = 69)	(*n* = 114)	(*n* = 362)
Age (years)	Mean (SD)	29.4 (10.0)	36.9 (10.4)	15.2 (8.9)	29.6 (8.0)	27.6 (9.3)	27.3 (6.6)	25.1 (10.3)
	Min–Max	18.0–58.0	22.0–54.0	6.0–40.0	19.0–46.0	18.0–54.0	18.0–40.0	6.0–58.0
Gender (*n* [%])	Female	5 (25.0)	10 (66.7)	33 (35.5)	24 (47.1)	29 (42.0)	49 (43.0)	150 (41.4)
	Male	15 (75.0)	5 (33.3)	60 (64.5)	27 (52.9)	40 (58.0)	65 (57.0)	212 (58.6)
Age group (*n* [%])	<12	0 (0.0)	0 (0.0)	39 (41.9)	0 (0.0)	0 (0.0)	0 (0.0)	39 (10.8)
	12–17	0 (0.0)	0 (0.0)	26 (28.0)	0 (0.0)	0 (0.0)	0 (0.0)	26 (7.2)
	≥18	20 (100.0)	15 (100.0)	28 (30.1)	51 (100.0)	69 (100.0)	114 (100.0)	297 (82.0)
Smoking status (*n* [%])	Smoker	4 (20.0)	0 (0.0)	0 (0.0)	5 (9.8)	18 (26.1)	1 (0.9)	28 (7.7)
	Nonsmoker	16 (80.0)	15 (100.0)	93 (100.0)	46 (90.2)	51 (73.9)	113 (99.1)	334 (92.3)
Asthma (*n* [%])	Yes	–	–	93 (100.0)	26 (51.0)	34 (49.3)	114 (100.0)	267 (73.8)
	No	–	–	0 (0.0)	25 (49.0)	35 (50.7)	0 (0.0)	60 (16.6)
GINA classification (*n* [%])	GINA I	–	–	0 (0.0)	14 (27.5)	18 (26.1)	–	32 (8.8)
	GINA II	–	–	76 (81.7)	12 (23.5)	16 (23.2)	–	104 (28.7)
	GINA III	–	–	17 (18.3)	0 (0.0)	0 (0.0)	–	17 (4.7)
	GINA IV	–	–	0 (0.0)	0 (0.0)	0 (0.0)	–	0 (0.0)
	No asthma	–	–	0 (0.0)	25 (49.0)	35 (50.7)	–	60 (16.6)
FEV_1_ or PEF (% of predicted value)	*n*	–	–	93	51	69	61	274
	Mean (SD)	–	–	93.1 (10.6)	101.8 (15.2)	104.2 (14.2)	98.7 (9.8)	98.8 (13.1)
	Min–Max	–	–	80.0–139.0	80.1–135.7	75.0–147.0	82.2–123.8	75.0–147.0
Duration of allergic rhinoconjunctivitis (years)	*n*	20	15	77	51	69	114	346
	Mean (SD)	5.8 (3.2)	21.1 (10.1)	5.7 (5.1)	12.5 (8.6)	8.6 (7.8)	8.2 (5.8)	8.8 (7.4)
	Min–Max	2.0–13.0	2.0–40.0	1.0–20.0	2.0–39.0	1.0–45.0	0.0–24.0	0.0–45.0

*GINA* Global Initiative for Asthma, *FEV*_*1*_ Forced Expiratory Flow in 1 sec, *PEF* Peak Expiratory Flow

### Safety sets

Subjects of the double-blind placebo controlled (DBPC) safety set who received at least 1 dose of the trial drug during the double-blind treatment period with 1800 PNU or placebo were combined (Trials AL1009ac, AL0104av, 97-09 M, 97-09 UK, AL0106ac, and AL0400av) in group HDM-DBPC (*n* = 214). The safety set HDM-ALL consists of patients from the DBPC phase plus patients from the asthma versus usual care trial (*n* = 279; Table 1).

Subgroups: The following subgroups were performed:

- Age group (<12, 12–17, ≥18 years)
- Gender (female/male)
- Subjects with asthmatic symptoms (Global Initiative for Asthma classification >0, GINA, 2006). Classification was assessed in Trials AL1009ac, AL0104av, AL0106ac, and AL0400av.

Spontaneous safety reports: For the period after launch of the product (01 July 2005) to 31 March 2016, ADRs were calculated in relation to the number of products sold and patients treated and classified according to expectedness. For unexpected ADRs, the system organ classes (SOCs) were reported.

### Coding of treatment-related AEs (TRAEs):

AEs were coded using the MedDRA version 15.0. AEs with onset during or after the first administration of the trial drug were defined as TRAEs. An AE was defined as being related to the trial drug if the causal relationship of the AE was assessed as “at least possible”.

## Results

### Clinical development

In all, 362 subjects (children, adolescents, adults) have received at least 1 dose of active treatment in the completed trials of the high-dose HDM allergoid clinical development program (Table 1)—114 subjects in the dose finding trial, 93 subjects in the allergic asthma trial, and 155 subjects in the allergic rhinitis/rhinoconjunctivitis trials. A total of 297 patients were treated with this HDM AIT during the DBPC period of the trials, 221 subjects with placebo. In children/adolescents, the trial drug was administered in an open-label design and usual care was used as control instead of placebo (*n* = 65).

### Marketed dose in clinical development

A total of 279 subjects received the marketed dose of 1800 PNU (0.6 ml strength B). The median number of injections administered in the 1800 PNU group was 15 injections during the 1^st^ treatment year (placebo: 15 injections), 12 injections during the 2^nd^ treatment year (placebo: 12 injections), and 8 injections during the 3^rd^ treatment year. The reasons for the difference in injection numbers are the up-dosing phase in year one and the possible prolongation of treatment intervals up to 8 weeks during maintenance treatment. In all, 148 patients received 1800 PNU for >2 years. Overall, 267 patients in the clinical development program of this HDM AIT had asthma: GINA I *n* = 32, GINA II *n* = 104, and GINA III *n* = 17 (114 patients without GINA classification). Patients had rhinoconjunctivitis for a mean of 8.8 (SD 7.4) years, min. 2–max. 45 years. A total of 65 patients were less than 18 years of age (only included in the asthma trial, Trial AL0104av): <12 years (*n* = 39), 12–17 years (*n* = 26; Table 1).

### Safety of marketed dose in clinical development

**Fig. 1:**
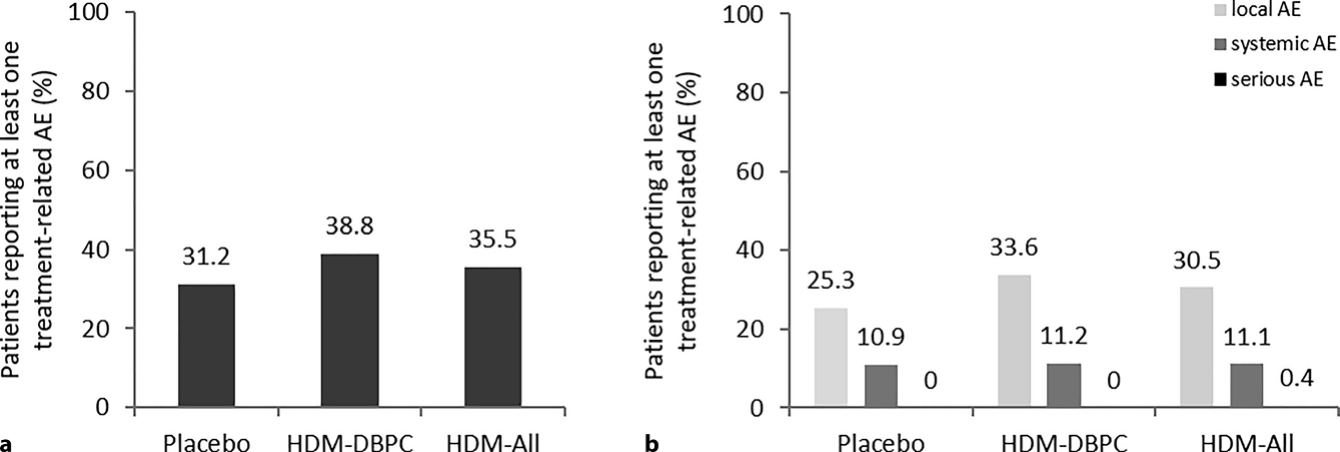
Percentage of patients reporting treatment-related adverse events related to study drug: **a** in the different treatment groups: placebo, HDM-DBPC, and HDM-All. **b** Distribution according to local, systemic and serious adverse event (AE). (DBPC Phase: placebo *n* = 221, HDM DBPC *n* = 214; HDM-All *n* = 279). *HDM-DBPC* high-dose HDM allergoid AIT double-blind placebo controlled, *HDM-RCT(UC)* high-dose HDM allergoid AIT usual care controlled, *HDM-ALL* HDM-DBPC plus HDM-RCT(UC), *AE* adverse event

In all, 214 patients received the 1800 PNU during the DBPC phase of the different trials and 65 in the asthma versus usual care trial (RCT(UC); Table 1), whereby 38.8% of patients treated with HDM AIT had an adverse event related to the trial drug, compared to 31.2% with placebo and 35.5% of the HDM AIT patients in the HDM-ALL group (Fig. 1). The difference in adverse events related to the study drug was primarily because of local reactions; there was no difference in the share of patients with systemic reactions (10.9% placebo, 11.2% HDM-DBPC, 11.1% HDM-All). One out of 279 patients in the HDM-All group experienced a serious adverse event (SAE): hospitalization because of erythema, conjunctivitis, asthma, cough, cyanosis, urticaria, and wheezing 15 min after administration of the trial drug during the 3^rd^ treatment year. The event resolved on the same day. Exacerbations of asthma were mentioned in the medical history. The patient was treated with an inhaled corticosteroid dose of 200 µg fluticasone as asthma controller medication that was reduced to 100 µg 4 weeks before the SAE. No SAEs were observed in placebo or HDM-DBPC group.

An overview about the TRAEs related to the trial drug and reported in >2% of patients is presented in Table 3.

**Table 3:** Treatment-related adverse events related to the trial drug reported in >2% of subjects by PT in decreasing frequency

	Number (%) subjects		
	DBPC Phase		DBPC plus RCT(UC)
	Placebo	HDM DBPC	HDM-All
	(*N* = 221)	(*N* = 214)	(*N* = 279)
*Subjects with related treatment-emergent AE*	*69 (31.2)*	*83 (38.8)*	*99 (35.5)*
Injection site swelling	51 (23.1)	61 (28.5)	71 (25.4)
Injection site pruritus	11 (5.0)	25 (11.7)	31 (11.1)
Injection site erythema	4 (1.8)	16 (7.5)	17 (6.1)
Injection site pain	5 (2.3)	9 (4.2)	15 (5.4)
Rhinitis	5 (2.3)	7 (3.3)	8 (2.9)
Pruritus	0 (0.0)	7 (3.3)	7 (2.5)
Cough	2 (0.9)	6 (2.8)	8 (2.9)
Sneezing	6 (2.7)	1 (0.5)	1 (0.4)
Rhinitis allergic	3 (1.4)	2 (0.9)	6 (2.2)
Fatigue	1 (0.5)	5 (2.3)	5 (1.8)

*DBPC* double-blind placebo controlled, ​*RCT(UC)* usual care controlled, *HDM-DBPC* high-dose HDM allergoid AIT double-blind placebo controlled, *HDM-RCT(UC)* high-dose HDM allergoid AIT usual care controlled, *HDM-ALL* HDM-DBPC plus HDM-RCT(UC), *AE* adverse event

Clinical laboratory measurements (biochemistry, hematology, and urinalysis) were analyzed in most of the trials. A shift of normal at baseline to abnormal after treatment was reported for >5% of subjects in any group only for eosinophils and neutrophils (14 subjects with HDM AIT). There were no other noteworthy differences between the groups.

### Subgroup analysis

**Fig. 2:**
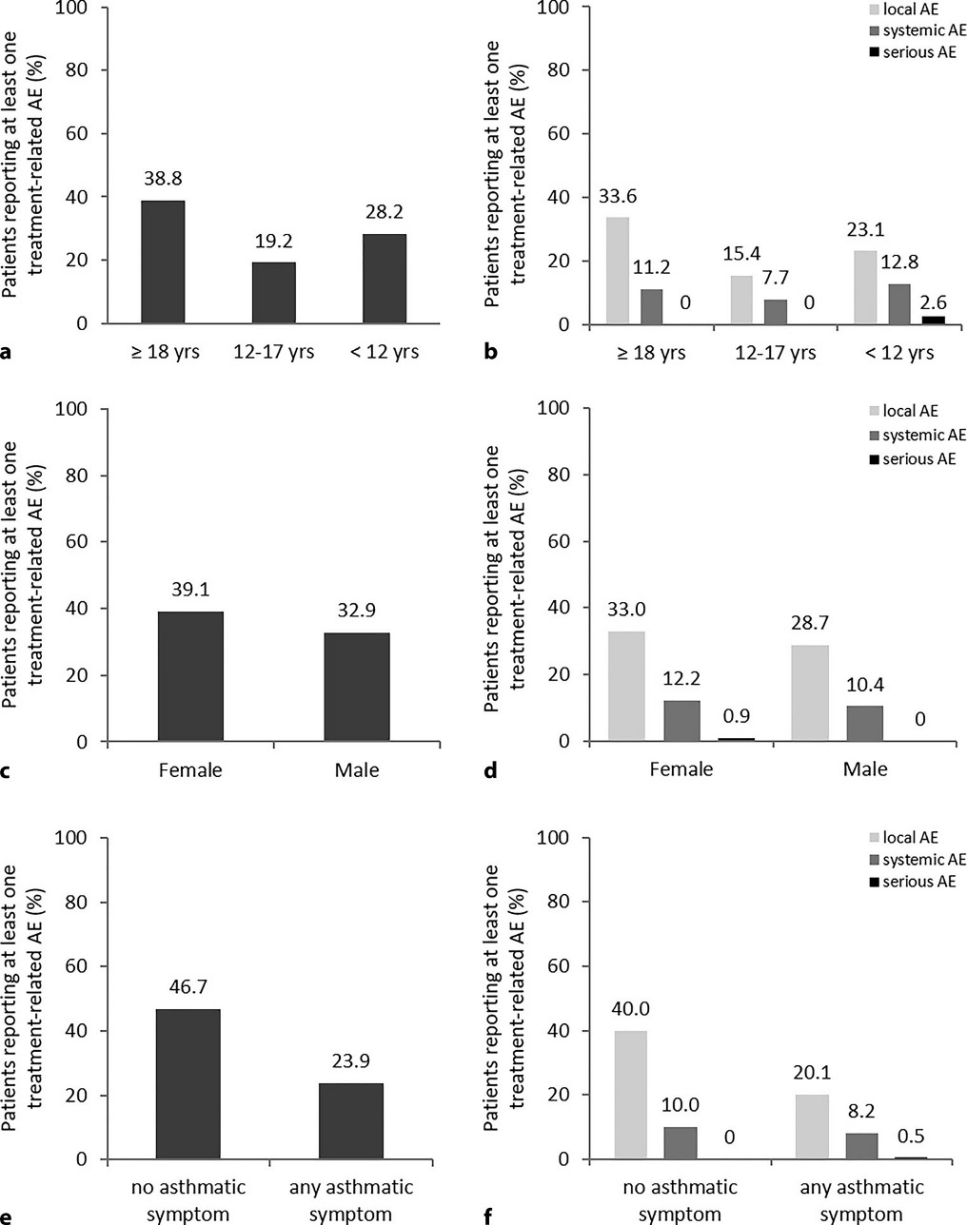
Percentage of patients reporting treatment-related adverse events related to study drug analyzed in different subgroups: age (**a,** **b**), gender (**c,** **d**), asthma status (**e,** **f**). **a** Data are shown for HDM-DBPC (≥18 years) and HDM-RCT(UC) (12–17 years and <12 years). **b** Distribution according to local, systemic and serious AE. **c** Percentage of female and male patients in HDM-All reporting a treatment-related AE. **d** Distribution according to local, systemic and serious AE. **e** Percentage of patients with and without asthma reporting a treatment-related AE and **f** Distribution according to local, systemic and serious AE. (Age (**a,** **b**): ≥18 years *n* = 214, 12–17 years *n* = 26, <12 years *n* = 39; Gender (**c,** **d**): female *n* = 115, male *n* = 164; Asthma status (**e,** **f**): no asthma symptom *n* = 60, any asthma symptom *n* = 184). *HDM-DBPC* high-dose HDM allergoid AIT double-blind placebo controlled, HDM*-RCT(UC)* high-dose HDM allergoid AIT usual care controlled, *HDM-ALL* HDM-DBPC plus HDM-RCT(UC), *AE* adverse event, *yrs* years

A summary of TRAEs by subgroups—age (a, b), gender (c, d), asthma status (e, f)—is presented in Fig. 2. Children and adolescents had fewer related adverse events and local reactions compared to adults; systemic reactions were slightly more frequent in children (12.8% versus 11.2% in adults), but much lower in adolescents (7.7%). Females had more adverse events in all 4 groups compared to males (see Fig. 2c, d). Treatment was well tolerated in asthmatic patients and the number of related adverse event, local reactions, and systemic reactions were low (see Fig. 2e, f), but one SAE occurred.

### Spontaneous safety reports

In the period from the first market launch (01 July 2005) to 31 March 2016 more than hundred thousand patients were treated with HDM AIT 1800 PNU as maintenance dose. ADRs were reported in 0.5% of patients compared to 38.8% (HDM-DBPC) and 35.5% (HDM-ALL) in clinical development; 94.6% of these ADRs were expected. The unexpected events were primarily reported in the SOCs: nervous system disorders (headache, hypoesthesia, paresthesia); musculoskeletal and connective tissue disorders (arthralgia, myalgia, pain in extremity); general disorders and administration site conditions (pyrexia); gastrointestinal disorders (oral mucosal blistering, nausea, gastrointestinal pain); and skin and subcutaneous tissue disorders (alopecia, eczema). No special patient group could be identified to be at higher risk, and no safety concern has arisen from this received post marketing safety data.

### Subgroup analysis of spontaneous safety reports

In the period from the first market launch to 31 March 2016 several ten thousand children and adolescents were also treated with HDM AIT 1800 PNU. ADRs were reported in 0.9% of children (0–11 years of age), 0.6% of adolescents (12–17 years) compared to 0.3% in adults (≥18 years).

If we assume that 50% female and 50% male patients were treated after launch, the number of ADRs were comparable irrespective of gender (female 0.4%, male 0.5%). The same was true for asthma. Asthma is found in up to 38% of patients with allergic rhinitis [11]. With this HDM AIT SCIT 0.4% of ADR reports were in patients with asthma and 0.5% in non-asthmatics.

## Discussion

In recent years, publications on HDM immunotherapy focused on sublingual treatment. Several multinational, randomized, placebo-controlled clinical trials of high quality standards in different patient populations and subgroups are available. In addition, this treatment route is known to be safe, which is why we compared our data with relevant data of sublingual products recently published in international literature.

### Rhinoconjunctivitis

In our observation of patients with rhinoconjunctivitis with/without asthma, adverse events with at least possible relationship to the high-dose HDM allergoid were 38.8% compared to 31.2% with placebo. This is lower than the, at least possibly, related adverse events seen in allergic rhinitis patients treated with HDM SLIT tablet (48.0% 6 SQ-HDM, 53.0% 12 SQ-HDM, 15.0% placebo; [12]; Table 4). The related AEs were primarily local reactions (33.6%) in HDM SCIT (injection site swelling 28.5%, -pruritus 11.7%, -erythema 7.5%, -pain 4.2%). This is comparable to the safety profile of HDM SLIT where the most common AEs are reported as being related to the investigational medicinal product (IMP) by the investigator were oral pruritus, throat irritation, and mouth edema (20.0, 14.0, and 8.0% of subjects on active treatment, respectively; [12]). In a recently published double-blind trial, evaluating the efficacy and safety of HDM tablets in 968 adolescent and adult patients (aged 12–64 years) with HDM-induced AR with or without intermittent asthma over 52 weeks, the number of subjects with any adverse drug reaction was 66.8% with the recommended dose 300 IR (73.1% with 500 IR), and 18.6% with placebo [13]. In two pooled RC clinical trials with 1215 subjects (12 SQ HDM tablet *n* = 600, 615 placebo) with a clinical history of HDM allergic rhinitis, and 863 (71%) with additional HDM allergic asthma 50% of subjects on active treatment reported TRAEs  compared to 16% of subjects on placebo [14].

**Table 4:** Safety overview of the high-dose HDM allergoid versus SLIT mite tablets

Product	ADRs with at least possible relationship to study drug (%)		
	Rhinoconjunctivitis ± Asthma	Asthma	Pediatrics with asthma (12–17 years)
*HDM allergoid*			
1800 PNU high-dose HDM allergoid	38.8	23.9	19.2
Placebo	31.2	18.0	–
*HDM SLIT tablet* [12, 14–16]			
6 SQ-HDM	48	39	55
12 SQ-HDM	53/50	46	50
Placebo	15/16	17	32
*HDM SLIT tablet* [13]			
300 IR	66.8	–	–
500 IR	73.1	–	–
Placebo	18.6	–	–

*ADR* adverse drug reactions, *PNU* protein nitrogen units, *HDM* house dust mites, *SLIT* sublingual immunotherapy

### Asthma

HDM is the most common allergen associated with asthma and more than 40% of adult asthmatics are atopic with a positive skin prick test result for the HDM allergens [15]. Adverse events related to the high-dose HDM allergoid were 23.9% in the group of patients with asthma compared to 46.7% in patients without asthma, placebo 18%; subjects with TRAEs were 47.9% compared to 51.6% with placebo. In a recently published study of asthma patients with a sublingual HDM tablet, AIT TRAEs occurred in 39.0% in the 6 SQ-HDM group, 46.0% in the 12 SQ-HDM group, and 17.0% with placebo [14]. A possibly treatment-related serious adverse event was reported for the 12 SQ-HDM tablet: asthma (moderate, alternative etiology was “recently viral infection”; [15]). With the high-dose HDM allergoid a serious adverse event (erythema, conjunctivitis, asthma, cough, cyanosis, urticaria, wheezing) was reported in an asthmatic child 15 min after administration of trial drug. Exacerbations of asthma were mentioned in the medical history and the corticosteroid dose was reduced from 200 µg fluticasone to 100 µg 4 weeks before the SAE. These examples show how important it is that asthma is controlled, especially when an AIT is performed and guidelines are followed.

### Pediatrics

There is little data available about the safety of HDM AIT in children and adolescents. In a recently published multicenter, double-blinded, randomized trial in adolescents (12–17 years old) with HDM allergic rhinitis with and without conjunctivitis and with or without asthma, patients received placebo, HDM SLIT tablet 6 SQ or 12 SQ once daily for 28 days. The proportion of subjects who experienced TRAEs was 25.0%, 45.0%, and 52.0% for the placebo, 6 SQ-HDM, and 12 SQ-HDM groups, respectively [16]. With the high-dose HDM allergoid, TRAEs were observed in 19.2% of adolescents (12–17 years old). There was a greater incidence of TRAEs in adolescents with asthma receiving the HDM SLIT tablet compared with placebo (asthma: 32.0%, 55.0%, 50.0%, placebo, 6‑SQ, 12-SQ respectively; without asthma: 21.0%, 40.0%, 54.0%; [16]). In the high-dose HDM allergoid group of adolescents, all had asthma and received the trial drug for up to two years (28 days for HDM SLIT).

### Gender

No literature is available about safety differences of HDM immunotherapy in females versus males. In the clinical development program of the HDM SCIT allergoid we observed a few more related AEs in females (39.1%) compared to males (32.9%), but more data are needed.

### Spontaneous safety reports

The safety of a medicinal product after launch in daily practice with much higher numbers of treated patients and a broad range of different patients treated adds to the safety information of a product. Very rare side effects might be seen for the first time and unexpected side effects might be reported. With the HDM SCIT product presented in this publication, adverse drug reactions were reported in 0.5% of patients compared to 38.8% (HDM-DBPC) and 35.5% (HDM-ALL) in clinical development, whereby 94.6% of these ADRs were expected, and there was no major concern about the unexpected ADRs related to the safety of this SCIT product in daily routine.

### Pediatric data analyzed from spontanous safety reports

Compared to adults slightly more children and adolescents (0.9% children, 0.6% adolescents, 0.3% adults) experienced an ADR. This is based on a great database of several thousand treated patients and possibly a higher reporting rate of ADRs in younger age groups. No new safety information has arisen concerning system organ classes or preferred terms of the reported ADRs.

### Gender data analyzed from spontanous safety reports

According to post marketing safety data no gender specific safety difference could be identified (female 0.4%, male 0.5%), and the high-dose HDM allergoid is well tolerated in asthmatic as well as non-asthmatic patients (0.4% asthma, 0.5% non-asthma patients with ADR).

On the basis of more than hundred thousand treated patients with the high-dose HDM allergoid in daily routine and a broad range of different patient groups, no special patient group could be identified to be at higher risk, no safety concern has arisen from the unexpected ADRs and the safety was comparable to what was already known from clinical development.

## Conclusion

Subcutaneous treatment with the high-dose HDM allergoid was well tolerated in HDM respiratory allergic disease in clinical development. The observed difference to placebo was primarily driven by local reactions; there was no difference in systemic reactions. The high-dose HDM allergoid was also well tolerated in daily practice. As 95% of ADRs were expected, no safety concern has arisen from the post marketing safety data. The product was well tolerated in all age groups (children, adolescents, and adults), in asthmatics and non-asthmatics. Furthermore, the safety profile was comparable to HDM SLIT tablets.

## Abbreviations

AA: Allergic asthma
ADR: Adverse drug reaction
AE: Adverse event
AIT: Allergen immunotherapy
AR: Allergic rhinitis
ARD: Allergic respiratory disease
DBPC: Double-blind placebo controlled
FEV_1_: Forced expiratory flow in 1 sec
GINA: Global Initiative for Asthma
HDM: House dust mite
HDM-ALL: HDM-DBPC plus HDM-RCT(UC)
PEF: Peak expiratory flow
PL: Placebo
PNU: Protein nitrogen units
RCT: Randomized, controlled trials
SAE: Serious adverse event
SCIT: Subcutaneous immunotherapy
SLIT: Sublingual immunotherapy
SOC: System organ class
TRAE: Treatment-related adverse event
UC: Usual care
